# No two are alike: on the role of *Klebsiella pneumoniae* permeability barriers in antibiotic susceptibility and persistence

**DOI:** 10.1128/aac.00085-25

**Published:** 2025-06-17

**Authors:** Inga V. Leus, Helen I. Zgurskaya

**Affiliations:** 1 Department of Chemistry and Biochemistry, University of Oklahoma 522128 , Norman, Oklahoma, USA; Shionogi Inc., Florham Park, New Jersey, USA

**Keywords:** *Klebsiella pneumoniae*, antibiotic resistance, multidrug efflux, outer membrane

## Abstract

*Klebsiella pneumoniae* and *Escherichia coli* cause a broad range of human infections with multidrug-resistant strains presenting serious therapeutic challenges in clinics. The discovery of new antibiotics effective against these pathogens is hindered because of effective drug permeability barriers comprising active efflux pumps acting synergistically with the low-permeability outer membrane. In this study, we characterized these barriers in *K. pneumoniae* American Type Culture Collection (ATCC) 43816, a hypervirulent strain broadly used in infection models. For this purpose, we constructed an efflux-deficient strain lacking the outer membrane channel TolC, which is required for activities of most resistance-nodulation-division efflux pumps, a hyperporinated strain producing a large non-specific pore in the outer membrane, and a double-compromised strain combining these two features. We next compared the contributions of the permeability barriers of ATCC 43816 to those of the laboratory *E. coli* BW25113 in antibiotic susceptibilities and persistence and in the intracellular accumulation of fluorescent probes. We identified significant differences between these two related species. Our results show that the outer membranes of *K. pneumoniae* and *E. coli* differ in their permeability to fluorescent probes and antibiotics and that *K. pneumoniae* is more effective in efflux of various compounds. We further found that nutrient-rich and minimal growth media do not affect permeation of all antibiotics in the same way and that the mechanism of antibiotic action and specific physicochemical properties of each compound play a defining role. Likewise, the roles of permeability barriers in the persistence of *K. pneumoniae* and *E. coli* vary, depending on the mechanism of antibiotic action, its external concentration, and the affected barrier.

## INTRODUCTION

Gram-negative pathogens rapidly spread in clinics and present significant therapeutic challenges. Among these pathogens, carbapenem-resistant *Enterobacteriaceae* top the Centers for Disease Control’s and World Health Organization’s lists of urgent pathogens (1, 2). These pathogens, like other gram-negative bacteria, protect themselves from small molecule toxins by elaborate cellular envelopes consisting of a low-permeability barrier of the outer membrane (OM), the inner membrane (IM), and active efflux pumps acting across both membranes (3, 4). The OM is an asymmetric bilayer of lipopolysaccharides (LPS) and phospholipids. In general, the outer LPS leaflet hinders the permeation of hydrophobic and large molecules, whereas small amphiphilic molecules can penetrate through non-specific porins that enterobacteria use for uptake of nutrients (5). The IM has orthogonal permeability properties and protects cells from hydrophilic and charged molecules (6).

Molecules penetrating the membranes are intercepted by various active efflux pumps acting synergistically with the diffusion barriers and expelling drugs from the cytoplasm, periplasm, or both (7). The most efficient efflux pumps are secondary drug:proton antiporters of the resistance-nodulation-cell division (RND) superfamily (8, 9). RND pumps transport drugs across the OM by engaging in protein complexes with a periplasmic membrane fusion protein and an outer membrane factor channel (10, 11). Together, the three components span the two membranes and the periplasm and can capture drugs from the cytoplasm, the inner membrane, or the periplasm.

We and others have previously shown that gram-negative pathogens differ dramatically in their permeability to various classes of antibiotics (12–14). Analyses of antibacterial activities and intracellular accumulation using strains with variable permeability barriers have shown that the OM barrier plays the dominant role in the protection of *Escherichia coli* (Ec) and *Pseudomonas aeruginosa*, but efflux defines the permeability of *Acinetobacter baumannii* cells (12). These three species differ dramatically in the lipid and porin compositions of their outer membranes as well as in the number and properties of efflux pumps encoded in their genomes. Furthermore, we found that even compounds of the same chemotype may use different permeation pathways, depending on small chemical modifications to gain access to intracellular targets. Accordingly, a classification analysis revealed limited conservation of molecular properties that define compound penetration into these bacteria (12, 15). In addition, antibiotic class-specific mechanisms of resistance such as modifying or degrading enzymes present in clinical isolates can act synergistically with both the OM and efflux barriers (7).

In this study, we analyzed the permeability barrier of *Klebsiella pneumoniae* (Kp) and compared it to that of *E.- coli*. These two related species of *Enterobacteriaceae* co-exist in the same environment of human gut and present a serious threat in clinics due to the emergence of multidrug and carbapenem-resistant strains (1). *E. coli* and *K. pneumoniae* share the overall organization of their cell envelopes. The major non-specific porins OmpF/OmpC in *E. coli* and OmpK35/OmpK36 in *K. pneumoniae* mediate the import of small hydrophilic molecules, including the influx of antibiotics such as β-lactams (Table S1) (16, 17). Clinical isolates often reveal reduced expression for one or both porins, suggesting that hampered influx contributes to bacterial resistance (16, 18, 19). Moreover, mutations in porins play a role in antibiotic resistance (20–23). In addition to the major porins, *K. pneumoniae* expresses other porins associated with compound influx. For example, the anion-specific PhoE porin is able to restore antibiotic susceptibility in the double OmpK35/K36 knockout strain, suggesting a path for entry (18).

Both *E. coli* and *K. pneumoniae* produce the major constitutive RND efflux pump AcrAB-TolC, which is 91% identical in the two species, according to sequence alignments (data not shown). While the mutational overexpression of AcrAB-TolC is typically associated with clinical antibiotic resistance in *E. coli* (24–26), several RND pumps were identified as contributors to resistance in *K. pneumoniae* (Table S2). In addition to AcrAB-TolC, the overproduction of KexD (not present in *K. pneumoniae* American Type Culture Collection [ATCC] 43816), OqxAB, and EefABC was also reported in antibiotic-resistant isolates of *K. pneumoniae* (27–30). The relative contributions of active efflux and the outer membrane barrier to antibiotic non-susceptibility of *K. pneumoniae* remain unclear.

To compare the permeability barriers of *E. coli* and *K. pneumoniae*, we applied a previously developed approach that separates contributions of the OM and efflux in susceptibilities to antibiotics and in their intracellular permeation (31). For this purpose, we constructed and used a set of four Kp strains with variable efflux and OM barriers: the wild-type ATCC 43816 (Kp-WT), its efflux-deficient Δ*tolC* derivative (Kp-E), the hyperporinated Kp-P strain producing a large non-specific pore in the OM, and the double-compromised Kp-PE strains lacking the major efflux pump and carrying the hyperporinated OM. We characterized and compared the contribution of each barrier in susceptibilities of *K. pneumoniae* and *E. coli* to various antibiotics in rich and minimal media to kinetics of intracellular accumulation of fluorescent probes and to antibiotic persistence.

## RESULTS

### *K. pneumoniae* ATCC 43816 is resistant to β-lactams and other antibiotics due to synergy between efflux and outer membrane barrier

For this study, we selected the ATCC 43816 (Kp-WT thereafter) strain, which is commonly used to study *K. pneumoniae* pathogenesis in animals (Table 1) (32). This strain is hypervirulent in several infection models and produces capsule and the two major efflux pumps AcrAB-TolC and OqxAB-TolC (33). Analysis of the susceptibility of Kp-WT to selected representatives of antibacterial agents showed that the strain is resistant to most antibiotics, including β-lactams (Table 2).

**TABLE 1:** Strains and plasmids used in this study^*a*^

Strain or plasmid	Relevant genotype	Source
*K. pneumoniae* ATCC 43816 (Kp)	Wild type	ATCC
IL203 (Kp-WT)	ATCC 43816 attTn7::miniTn7-Km^r^-*araC* -P*_araBAD_*-MCS	This study
IL204 (Kp-P)	ATCC 43816 attTn7::miniTn7-Kmr-araC-ParaBAD-fhuAΔC/Δ4L	This study
IL205	ATCC 43816 *ΔtolC*	This study
IL206 (Kp-E)	IL205 attTn7::miniTn7-Km^r^-*araC*-P*_araBAD_*-MCS	This study
IL207 (Kp-PE)	IL205 attTn7::miniTn7-Kmr-araC-ParaBAD-fhuAΔC/Δ4L	This study
GKCW101 (Ec-WT)	BW25113 *att*Tn*7*::mini-Tn*7*T-Km^r^-*araC*-P*_araBAD_*-MCS	(31)
GKCW102 (Ec-P)	BW25113 attTn7::mini-Tn7T-Kmr-araC-ParaBAD-fhuAΔC/Δ4L	(31)
GKCW103 (Ec-E)	GD102 attTn7::mini-Tn7T-Kmr-araC-ParaBAD-MCS	(31)
GKCW104 (Ec-PE)	GD102 attTn7::mini-Tn7T-Kmr-araC-ParaBAD-fhuAΔC/Δ4L	(31)
pTNS3	Ap^r^; Helper plasmid encoding Tn*7* transposase proteins TnsABCD from P1 and P*_lac_* promoter	(34)
pGK-R6K-mini-Tn*7*T-*araC*-P_BAD_-FhuAΔC/Δ4L (Km^r^)	pUC18T *ori*R6K mini-Tn*7*T Km^r^ *araC* P_BAD_, vector containing the *fhuA*Δ*C/*Δ*4L* gene	(31)
pGK-R6K-mini-Tn*7*T-*araC*-P_BAD_-MCS (Km^r^)	pUC18T *ori*R6K mini-Tn*7*T Km^r^ *araC* P_BAD_	(31)
pIL105	pMo130 plasmid containing gentamicin-resistance cassette, Gm^r^	(35)
pIL154	pMo*∆tolC*::Gm^r^ containing 0.9 kb upstream and 1 kb downstream fragments of *K. pneumoniae tolC*; Gm^r^	This study
pKD46	Ap^r^, λ red recombinase expression	(36)
pIL155 (pKD46-Km)	Km^r^, λ red recombinase expression	This study
pCP20	FLP^+^, λ cI857^+^, λ p_R_ Rep^ts^, Ap^r^, Cm^r^	(37)

^*a*^ Apr, Kmr, Gmr, and Cmr are genes encoding resistance to ampicillin, kanamycin, gentamicin, and chloramphenicol, respectively.

**TABLE 2:** Minimal inhibitory concentrations (μg/mL) of representative antibacterial agents against constructed *K. pneumoniae* strains measured in Luria-Bertani broth

Agent	Kp-WT	Kp-P	Kp−Ε	Kp-PE
Erythromycin	256	32	8	0.25
Azithromycin	32	2	1-2	0.0625
Sodium dodecyl sulfate	>1,024	>1,024	16	8
Zeocin	16	0.5	0.25	0.0625
Ciprofloxacin^*a*^	0.064 (0.032)	0.016 (0.008)	0.016 (0.004)	0.004 (0.004)
Rifampin	16	1	16	1
Ethidium	>128	>128	64	32
Novobiocin	64	1.25	1.25	0.16
Chloramphenicol	4–8	4	1	1
Gentamicin	8	8	4	2
Tobramycin^*a*^	2 (8)	2 (4)	2 (4)	1 (2)
Streptomycin	4	2	2	1
Trimethoprim	1–2	0.5	0.5	0.5
Carbenicillin	512	16–32	512	16–32

^*a*^ MICs of the analogous *E. coli* strains are shown in parentheses.

**Fig 1:**
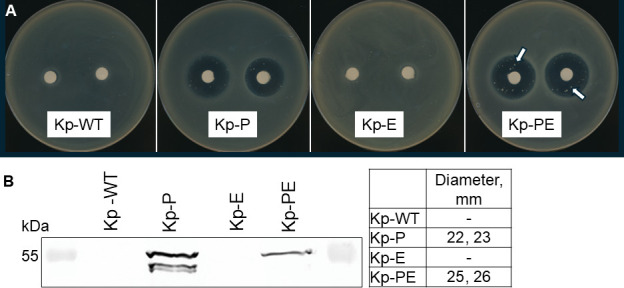
Activity and expression of the recombinant pore in *K. pneumoniae.* (A) Vancomycin spot assay. Arrows indicate spontaneous vancomycin-resistant mutants due to the inactivation of the pore. The quantification of the clearance zones is shown in the table below. (B) Immunoblotting analysis of the solubilized and partially purified OM proteins after 6 h induction with monoclonal anti-His tag antibody.

To modulate the permeability barrier of ATCC 43816, we integrated the gene encoding a large, engineered OM pore into the *K. pneumoniae* Tn7 site on the chromosome and under an L-arabinose-inducible promoter (Table 1). This pore (P) is a derivative of *E. coli* FhuA lacking the C-terminal domains and four external loops, which creates ~2.4 nm holes in the outer membrane (31). In agreement with previous studies, as seen from large zones of inhibition, the hyperporinated Kp-P cells became sensitive to killing by vancomycin, which is a large glycopeptide antibiotic that does not penetrate through the OM porins (Fig. 1A). Immunoblotting analyses of the purified outer membrane proteins confirmed the presence of the His-tagged pores (Fig. 1B). Thus, the pores are produced and functional in *K. pneumoniae*.

In addition to vancomycin, hyperporination strongly affected the activities of macrolides (MACs) erythromycin and azithromycin, zeocin, rifampin, novobiocin, and carbenicillin (Table 2). In contrast, no changes or only two- to fourfold reductions in minimum inhibitory concentrations (MICs) were found for aminoglycosides gentamicin, tobramycin and streptomycin, chloramphenicol, trimethoprim, detergent sodium dodecyl sulfate (SDS), and a fluorescent dye ethidium bromide (Et). Thus, the effect of OM hyperporination is the strongest for large and hydrophobic compounds exceeding the ~600 Da cutoff of non-specific porins.

To inactivate efflux, we constructed a Δ*tolC* strain (Kp-E). TolC is an outer membrane channel required for functions of all RND efflux pumps of *K. pneumoniae* except EefABC (Table S2) (27). Antibiotic susceptibility measurements showed that the effect of efflux inactivation was drug specific and in some cases exceeded the effect of hyperporination, suggesting that the activities of certain compounds, such as SDS, ethidium, and chloramphenicol, are primarily affected by efflux (Table 2). In contrast, MICs of rifampin and carbenicillin that were strongly reduced by hyperporination remained unchanged in efflux-deficient cells.

Finally, we constructed a double-compromised hyperporinated and efflux-deficient Kp-PE strain (Table 1). Several antibiotics that are affected by both barriers, such as macrolides, zeocin, and novobiocin, became highly potent in the double-compromised strain, pointing to the synergistic interactions between OM and efflux in protection against these antibiotics (Table 2). Likewise, these antibiotics were found to be protected by the synergism between efflux and OM barrier in other gram-negative bacteria (31, 38).

We also noticed that in the pore-producing strains, a few colonies reproducibly appeared in the vancomycin clearance zones (Fig. 1A). We have isolated these vancomycin-resistant colonies of Kp-P and Kp-PE strains, PCR-amplified the pore-encoding genes, and sequenced them. In all resistant isolates, we found insertions, deletions, and point mutations disrupting the coding sequence of the pore. No mutations were found in the genes encoding OmpK35, OmpK36, and OmpK37 porins. Hence, the pore inactivation leads to vancomycin resistance in these strains.

### *K. pneumoniae* and *E. coli* differ in the kinetics of small molecule accumulation

To characterize the kinetic properties of the *K. pneumoniae* permeability barrier, we analyzed time-dependent changes in the intracellular accumulation of three fluorescent probes that are known substrates of efflux pumps and used broadly to analyze their activities (39–41). Hoechst 33342 (HT) and Et are the cytoplasmic reporters and fluoresce most when bound to DNA. N-phenyl-naphthylamine (NPN) is a hydrophobic membrane probe that fluoresces when bound to lipids. We compared the kinetics of probes permeation in the *K. pneumoniae* four-strain set to that in the analogous Ec set of strains (Table 1).

**Fig 2:**
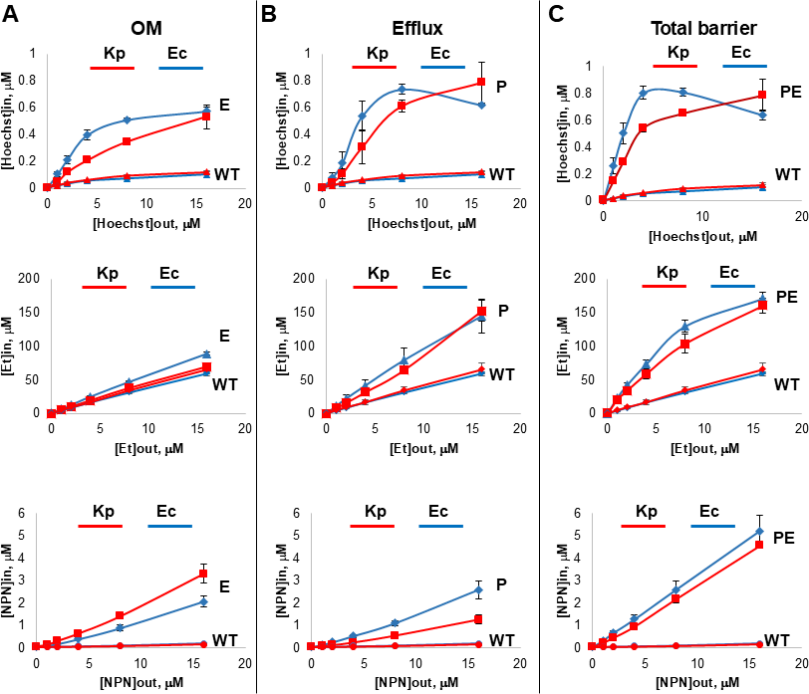
Steady-state intracellular accumulation levels of the known fluorescent substrates of efflux pumps. The four-strain sets of *K. pneumoniae* (red) and *E. coli* (blue) were incubated with increasing concentrations of Hoechst, ethidium bromide (Et), and NPN, and kinetics of the fluorescence change were monitored over a period of time. Collected kinetic curves were fitted into a model to extract the steady-state concentrations of probes inside the cells. These steady-state concentrations are plotted as functions of the external concentrations of the probes. Error bars are standard deviations of at least three independent and two technical repeats. WT, wild type. (A) Efflux-deficient (the OM permeability limits intracellular accumulation) cells. (B) Hyperporinated (efflux limits intracellular accumulation). (C) Double-compromised efflux-deficient and hyperporinated cells (remaining barriers).

Both Kp-WT and Ec-WT accumulated very low levels of all three probes during the experiment (Fig. 2). The initial jump in fluorescence is due to the binding of the probes to cell surfaces, which increases linearly with increasing concentrations of the probes and overlaps for the two species (40). The second step is a linear slow increase in fluorescence over the course of the experiment associated with the intracellular accumulation (Fig. S1 to S3).

In efflux-deficient Δ*tolC* strains, diffusion across the OM becomes the rate-limiting step (40). The comparison of steady-state levels of the intracellular accumulation of probes in Kp-E and Ec-E cells showed that the OM of *E. coli* is more permeable to HT and Et than *K. pneumoniae* OM, but the inverse was found for NPN, with Kp*-*E cells being more permeable to this hydrophobic probe (Fig. 2A**)**.

Hyperporination reduces the OM barrier, but efflux remains fully operational in such strains. Kp-P cells appear to be more efficient than Ec-P cells in the efflux of NPN and HT, as seen from higher steady-state accumulation levels in Ec-P cells (Fig. 2B). A similar trend can be seen in double-compromised PE strains, in which Ec-PE cells accumulated higher levels of HT than Kp-PE cells (Fig. 2C). 
However, steady-state accumulation levels of NPN and Et were very similar in both Ec-PE and Kp-PE strains.

Taken together, kinetic analyses showed that the OMs of two species have different permeability properties, whereas *K. pneumoniae* efflux appears to be more efficient than *E. coli* efflux.

### *K. pneumoniae* and *E. coli* differ in the major barriers for antibiotic permeation

**Fig 3:**
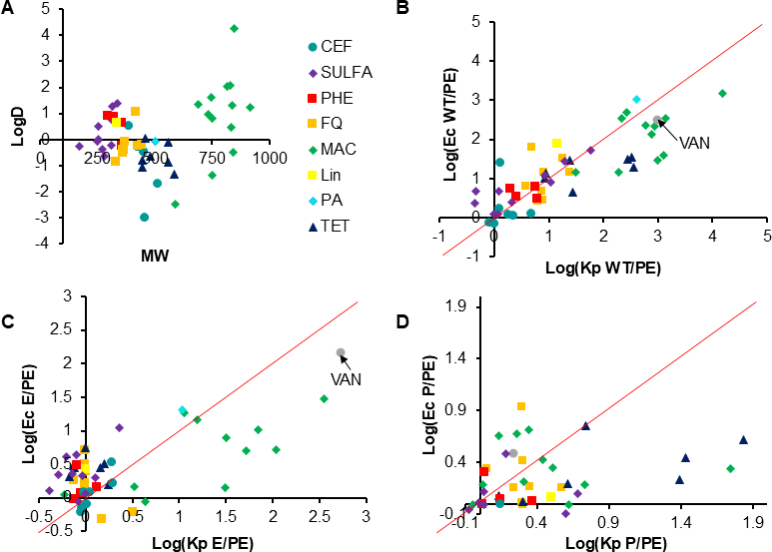
Antibiotic classes and their half maximal inhibitory concentration (IC_50_) ratios measured in M9 medium. (A) Molecular weight and LogD properties of analyzed antibiotics. (B) The plot of logarithms of total ratios of *E. coli* (Ec) and *K. pneumoniae* (Kp). (C) The same as panel B but for OM ratios. (D) The same as panel B but for efflux ratios. Lin, linezolid.

We previously described the roles of permeability barriers of three gram-negative bacterial species, including *E. coli*, in antibacterial activities and permeation of different classes of antibiotics (12). We next analyzed the same library of 43 antibiotics for antibacterial activities against the constructed *K. pneumoniae* four-strain set. The library includes 10 sulfonamides (SULFAs), 8 fluoroquinolones (FQ), 11 MACs, 7 tetracyclines (TET), 4 phenicols (PHE), pseudomonic acid, linezolid, and vancomycin (Fig. 3A; Table 3). In addition, we measured MICs of seven representative cephalosporins (CEFs). MICs were first analyzed in the M9-MOPS medium (12) because sulfonamides lack activities in Luria-Bertani (LB) broth.

**TABLE 3:** Minimal inhibitory concentrations (μM) of representative antibiotics measured in the minimal MOPS-M9 medium

Compound	Kp-PE	Kp-E	Kp-P	Kp-WT
Cefalonium	6.25	3.1–6.25	6.25	6.25
Cefazolin	6.25	3.1	3.1–6.25	3.1
Cefipime	0.031	0.031	0.031	0.063
Cefotaxime	0.008	0.016	0.008	0.063–0.125
Cefoxitin	6.25	6.25	3.1–6.25	12.5–25.0
Cefpirome	0.031	0.031	0.031–0.063	0.063
Cefprozil	12.5	12.5–25.0	12.5	25.0
Moxifloxacin	0.008–0.016	0.063	0.016–0.031	0.32–0.625
Ciprofloxacin	0.01	0.005–0.01	0.02–0.04	0.08–0.16
Levofloxacin	0.02	0.02	0.04	0.32
Fleroxacin	0.08	0.08	0.16	0.625
Clinafloxacin	0.005	0.016	0.01	0.063
Temafloxacin	0.02	0.02	0.04	0.625-1.25
Rufloxacin	0.625	0.32	0.625	5.0
Prulifloxacin	0.0125	0.0125	0.025	0.05–0.1
Doxycycline	0.05	0.05–0.1	0.4	3.2
Minocycline	0.1	0.1	1.6	25.0
Chlortetracycline	0.1–0.2	0.1	1.6	25.0
Tigecycline	3.2–6.25	3.2	6.25–12.5	50.0
Omadacycline	0.2–0.4	0.4	1.6	6.25–12.5
Eravacycline	0.125	0.125–0.25	0.25	1.0
Tetracycline	0.8	0.4–0.8	1.6–3.2	3.2
Dirithromycin	0.2	0.8	0.4	50.0
Roxithromycin	1.0	12.5–25.0	0.8–1.6	>100.0
Spiramycin	0.4	6.25–12.5	0.8	>100.0
Josamycin	0.1–0.2	6.25–12.5	1.6	>100.0
Tylosin	0.5	25–50	0.8	>100.0
Midecamycin	0.05	6.25	1.6	>100.0
Clarithromycin	0.16	3.2–6.25	0.63	>100.0
Solithromycin	0.02–0.04	3.2–6.25	0.16	50.0
Oleandomycin	3.2	6.25–12.5	6.25	>100.0
8-Fluoro erythromycin	0.1	3.2	0.2	>100.0
Descladinose azithromycin	1.6	3.2	1.6	100.0
Florfenicol	3.2	3.2	3.2	12.5
Thiamphenicol	50.0	25.0–50.0	50.0	100.0
Azidamfenicol	25–50	25.0	50.0	100.0
Chloramphenicol	3.2	3.2	6.25	12.5
Pseudomonic acid	0.08	3.2	0.32	100.0
Linezolid	25.0	25.0	≥100	>100.0
Vancomycin	0.16	100.0	0.16	>100.0
Sulfamethoxazole	1.6	1.6	1.6	1.6
Sulfadiazine	3.2	3.2	3.2	3.2
Sulfadimethoxine	1.6	1.6	3.2	25.0
Sulfadoxine	3.2	3.2	3.2–6.25	25.0
Sulfamethizole	6.25	6.25	6.25	6.25
Sulfathiazole	3.2	3.2	3.2	3.2
Sulfanilamide	>100.0	>100.0	>100.0	>100.0
Sulfamethopyrazine	3.2	1.6	6.25	12.5
Sulfaphenzole	0.8	1.6	3.2–6.25	100.0
Sulfanitran	25.0	50.0	>100.0	>100.0

All four strains of *K. pneumoniae* were susceptible to CEF, FQ, and some TET (Table 3). The MICs of CEF were affected neither by hyperporination nor by efflux inactivation, suggesting that these antibiotics efficiently permeate *K. pneumoniae* cells. The notable effect of hyperporination (8- to 16-fold) was seen only for cefotaxime. Likewise, with a few exceptions, the MICs of SULFA are not affected by hyperporination of the OM and efflux inactivation. Vancomycin, pseudomonic acid, and most of MAC were more strongly affected by hyperporination than by the loss of efflux, suggesting that for these large antibiotics, permeation across the OM is the major barrier for activities. In contrast, the activities of TET were primarily affected by efflux, as seen from the strong reduction in MICs against the Kp-E strain. Finally, the effect of permeability barriers on FQ activities is mixed, with MICs of some compounds equally reduced in Kp-E and Kp-P strains, whereas others were more strongly affected by efflux inactivation. The double-compromised Kp-PE strain was hypersusceptible to all tested antibiotics.

The contributions of permeation barriers were next assessed from ratios of the calculated IC_50_ values expressed as efflux (P/PE), outer membrane (E/PE), and the total barrier (WT/PE) ratios and compared to those of *E. coli* reported earlier (Table S3) (12). We found that the Kp WT/PE ratios were higher than those of *E. coli* against almost all macrolides, four out of seven TETs (doxycycline, tigecycline, minocycline, and chlortetracycline) and two FQs (moxifloxacin and clinafloxacin) (Fig. 3B). This result points to a more robust permeability barrier against these antibiotics in *K. pneumoniae.* In contrast, the activities of cefpirome, sulfamethoxazole, linezolid, and fleroxacin were more strongly affected by the *E. coli* barriers. For all other antibiotics, the two species were very similar to each other.

As seen from the comparison of the OM ratios (E/PE), the differences in MAC susceptibilities between the two species are largely due to a stronger OM barrier in *K. pneumoniae* (Fig. 3C). On the other hand, the *E. coli* OM presented a more formidable barrier for SULFA, TET, and several FQ representatives. Surprisingly, the contribution of efflux as seen from the activities of antibiotics was species- and drug-specific, reflecting the differences in efflux pumps between the two species (Fig. 3D). *K. pneumoniae* efflux was more effective against MAC midecamycin, solithromycin and josamycin, TET chlortetracycline, minocycline and doxycycline, and FQ ciprofloxacin. The *E. coli* efflux was stronger against FQ fleroxacin and MAC clarithromycin (Table S1). No obvious structural properties of these antibiotics could explain these differences in efflux specificities.

### Growth medium affects permeability barriers of both species in antibiotic-specific manner

**Fig 4:**
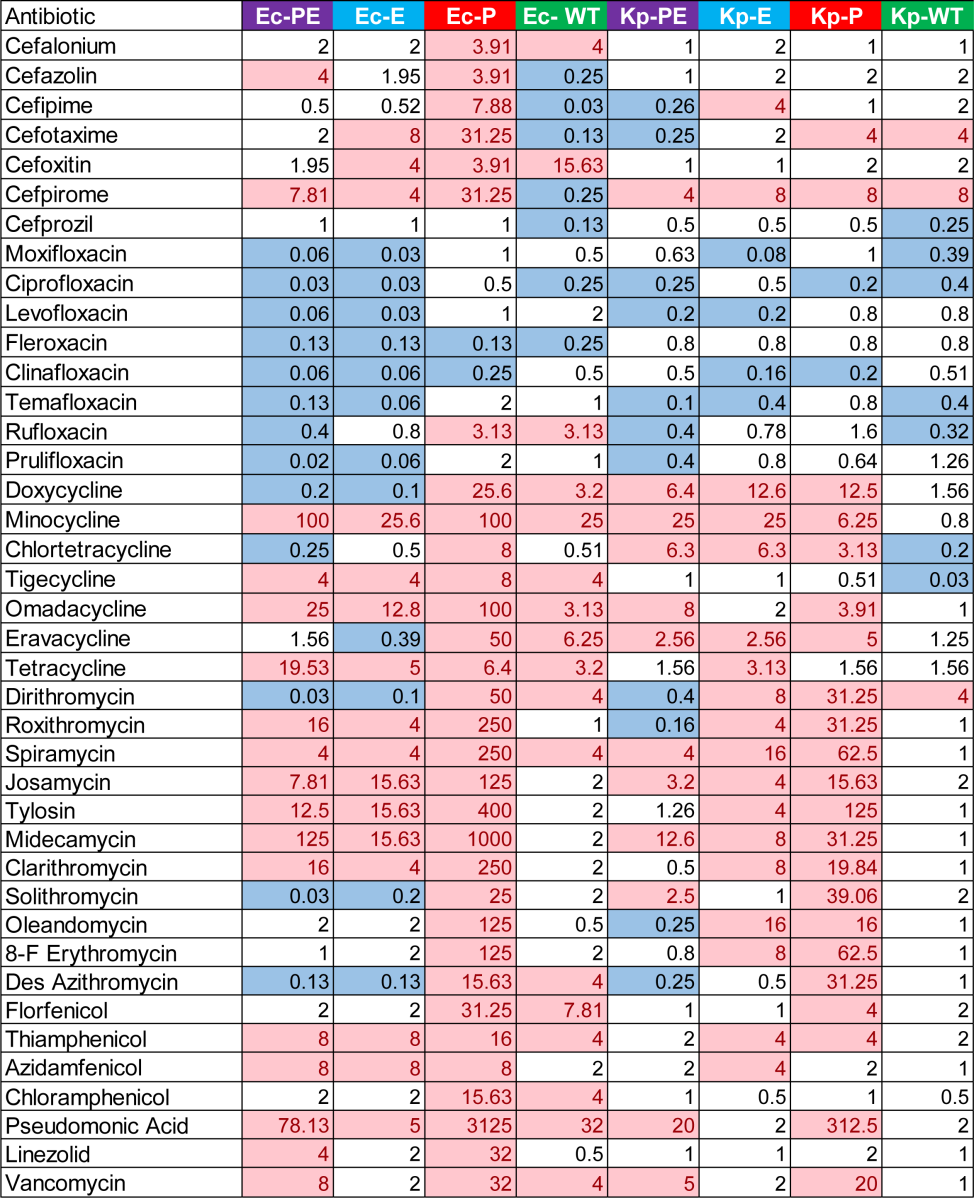
Fold change differences in MIC values measured for the indicated strains in the LB and M9 media. Values highlighted in blue are those with MICs more than twofold higher in M9, and those in red are those with MICs more than twofold higher in LB broth.

The minimal and rich media are commonly used in assessing potencies of antibacterial agents, and it is well recognized that the MICs of some antibiotics differ when analyzed in different growth media (42–44). Furthermore, a nutritional downshift in the minimal medium is one of the stresses bacteria experience during infections (45). For all antibiotics except SULFA, we next measured MICs in LB broth (Table S3), compared them to the MIC values obtained in M9, and calculated the LB/M9 ratios of the measured MICs (Fig. 4). When antibiotics were more efficient in the rich LB medium, the MICs in the minimal M9 medium were higher than those in the LB medium, and the calculated LB/M9 ratios were below 1. The LB/M9 ≤0.5 was deemed significant because we used the twofold dilution method to determine the MIC values. Correspondingly, the ratios above 1 (≥2 is significant) were observed when antibiotics were more effective in the M9 medium.

We found that most CEF activities against Kp-WT, as well as its barrier-compromised derivatives, were not sensitive to growth media, but those compounds affected by media were more active in the M9 medium. In contrast, most CEFs were more effective against Ec-WT in the LB medium. The exceptions are cefalonium and cefaxitin, both containing thiophene moiety, that were more effective in M9. Interestingly, the trend reversed in Ec-P cells, with all CEFs, except cefprozil, being more effective in the M9 medium (Fig. 4). This result suggests that the differences between activities of compounds against Ec-WT are due to changes in the permeability properties of the OM. The effect of efflux inactivation in Ec-E and Ec-PE was compound specific, but the activities of the affected compounds were enhanced in M9.

Most FQs were more effective against both Kp-PE and Ec-PE in the LB medium (Fig. 4). Interestingly, the growth medium-dependent changes in MICs of FQ against the wild type (WT) and the single barrier mutants of *E. coli* and *K. pneumoniae* were compound specific, suggesting that growth medium-dependent changes in their intracellular penetration are not specific to the active core of these antibiotics but rather are defined by specific chemical modifications.

All TETs (except chlortetracycline) were more effective against Ec-WT in the M9 medium. In contrast, only chlortetracycline and tigecycline were affected by the growth medium but were more effective against Kp-WT cells in the LB medium, whereas the rest of TET had similar MICs in both media (Fig. 4). Most TETs were found to be more effective in the M9 medium against hyperporinated Ec-P and Kp-P (Fig. 4). The effect of efflux inactivation on TET activities was species specific. Kp-E cells were more susceptible to TET when growing in M9, whereas the medium effect was compound specific for Ec-E. This finding suggests that the observed changes in the MICs of TET are due to the differences in specific permeability barriers of *E. coli* and *K. pneumoniae*.

For MAC (except dirithromycin), PHE, and other tested antibiotics, we found no differences in MICs against Kp-WT grown in the LB and M9 media. In contrast, Ec-WT cells were more susceptible to some of these antibiotics when grown in the M9 medium. Hyperporination dramatically enhanced this effect, as seen from further increase in LB/M9 MICs ratios for Ec-P cells as well as for Kp-P cells. The effects of growth media for efflux-deficient and double-compromised cells were more complex with both the species- and compound-specific differences.

To characterize the effect of growth media on the permeability barriers of *E. coli* and *K. pneumoniae*, we calculated the total barrier, efflux, and OM ratios of IC_50_ values and analyzed correlations between these ratios determined for all antibiotics in the M9 and LB media. We found that for both species, Pearson correlation coefficients were 0.1 for the total barriers WT/PE and were close to 0 for the efflux barrier P/PE, suggesting low and no correlation between M9 and LB media. Likewise, low-to-moderate correlations for both *E. coli* (*r* = 0.42) and *K. pneumoniae* (*r* = 0.34) were found between OM E/PE ratios in LB and M9 media.

Thus, the nutritional differences between M9 and LB media did not affect the permeability barriers in the same way for all tested antibiotics, and the effects were both antibiotic specific and bacterial species specific.

### *E. coli* and *K. pneumoniae* differ in antibiotic persistence

Antibiotic persisters are a small population of bacterial cells that are tolerant to killing by antibiotics (46, 47). The role of permeability barriers in antibiotic persistence remains unclear. We next analyzed how active efflux and OM barriers contribute to antibiotic persistence in *K. pneumoniae* and *E. coli*. We chose ciprofloxacin and tobramycin, both with bactericidal mechanisms of action but different mechanisms of action and permeation properties. Ciprofloxacin kills cells by inhibiting the type 2 DNA topoisomerases (48). This antibiotic is affected by both efflux and OM, and its MICs vary between the hyperporinated and efflux knockout strains by 8- to 16-fold (Table 2). Tobramycin inhibits protein translation and disrupts the integrity of cellular membranes (49). Unlike ciprofloxacin, tobramycin mostly avoids efflux and permeates the OM, as seen from the MIC ratios within two to fourfold between the four-strain sets of *K. pneumoniae* and *E. coli* (Table 2). To characterize persistence, stationary-phase cultures of the four-strain sets of each species were exposed to 10× MICs of the respective antibiotics, and CFUs were counted over a 24 h period.

**Fig 5:**
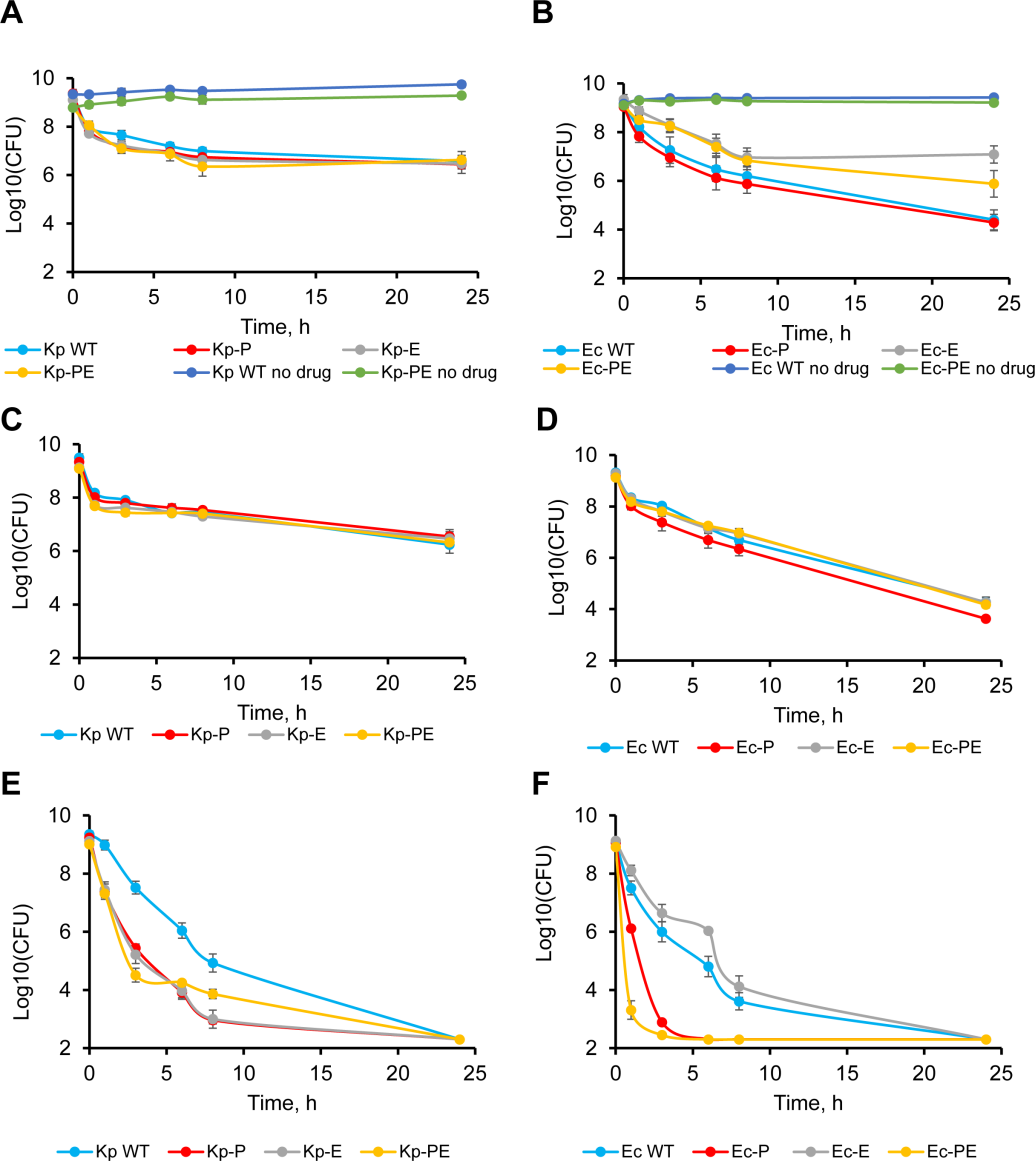
Ciprofloxacin and tobramycin persistence of stationary-phase planktonic cultures of *K. pneumoniae* and *E. coli.* Overnight cultures were washed and incubated with ciprofloxacin (**A through D**) or tobramycin (**E and F**). Cells were collected and washed at indicated time points, and CFUs were counted by plating onto LB agar plates. (A) The concentration of ciprofloxacin used was 10× MIC and specific for each *K. pneumoniae* strain (see Table 2). (B) Ciprofloxacin concentration was 0.32 µg/mL for Ec-WT, 0.08 µg/mL for Ec-P, and 0.04 µg/mL for Ec-E and Ec-PE. (C) The same concentration of ciprofloxacin 10× MIC of the Kp-WT (0.64 µg/mL) was used for all strains. (D) The same concentration of ciprofloxacin 10× MIC of Ec-WT (0.32 µg/mL) was used for all strains. (E and F). *K. pneumoniae* and *E. coli* strains were treated with 20 and 80 µg/mL, which corresponded to 10× MIC of Kp-WT and Ec-WT, respectively. Error bars are standard errors (*n* = 6–9).

In agreement with previous studies (50), we found that the kinetics of cell killing by both antibiotics is biphasic (Fig. 5). For Kp-WT cells treated with 10× MIC of ciprofloxacin, the quick kill phase lasted for 3–4 h, and the subsequent plateau indicated the persistent population (Fig. 5A). Surprisingly, neither efflux inactivation nor hyperporination of the OM changed this killing curve, suggesting that the permeability barriers do not play a critical role in *K. pneumoniae* persistence to ciprofloxacin (Fig. 5A).

The killing curve for Ec-WT was also biphasic. However, the quick phase eliminated at least 3 logs of CFUs, and the slow phase was continuous without reaching a plateau at 24 h, suggesting that cells were tolerant, but no persisters were present (Fig. 5B). Likewise, the hyperporinated Ec-P failed to form persisters, with CFU declining during the 24 h exposure to the 10× MIC of ciprofloxacin (Fig. 5B). The killing curves of efflux-deficient cells were different. For both Ec-E and Ec-PE cells, the quick phase was longer ~6 to 7 h and transitioned to a slow phase at ~1% of CFUs. Thus, efflux-deficient cells form persisters when exposed to 10× MICs of ciprofloxacin.

Since efflux-deficient *E. coli* strains are highly susceptible to ciprofloxacin with MICs in the 0.004 µg/mL range (Table 3), the differences in ciprofloxacin killing kinetics between the *E. coli* strains could be due to the different external concentrations of this antibiotic used in the experiment. We next analyzed killing kinetics for all four strains in the set at the same external concentrations of ciprofloxacin corresponding to the 10× MIC of ciprofloxacin against Kp-WT (0.64 µg/mL) and Ec-WT (0.32 µg/mL). We found that killing curves of ciprofloxacin were biphasic and overlapped for all four *K. pneumoniae* and *E. coli* strain sets (Fig. 5C and D). We conclude that different external concentrations of ciprofloxacin were responsible for differences in the killing kinetics of the efflux-proficient and efflux-deficient *E. coli* strains.

No changes in the killing curves were found for *K. pneumoniae* strains, suggesting that RND efflux and the OM permeability do not contribute to ciprofloxacin persistence even when the external concentrations of the antibiotic exceed the MICs by more than 100-fold (Fig. 5C). These results further show that MICs of ciprofloxacin determined for the growing cell cultures do not correlate with killing of the stationary-phase cells.

Unlike with ciprofloxacin, all *E. coli* and *K. pneumoniae* strains died 24 h after the exposure to the 10× MIC of tobramycin (Fig. 5E and F). In the case of *K. pneumoniae*, killing curves were biphasic for all strains, and the Kp-WT cells were the slowest (>8 h) to die (Fig. 5E). The biphasic curves of Kp-P, Kp-E, and Kp-PE strains were similar to each other, with about five orders of magnitude reduction in CFUs at 3–4 h after antibiotic exposure (Fig. 5E). Surprisingly, the CFUs of Ec-P and Ec-PE cells dropped below the detection limit at ~3 h after exposure to tobramycin (Fig. 5F). Killing kinetics of Ec-WT and Ec-E with the native OM was slower, but also no CFUs were detected at 24 h after exposure (Fig. 5F).

Taken together, our results show that the roles of permeability barriers in the persistence of *K. pneumoniae* and *E. coli* vary, depending on the mechanism of action of the antibiotic, its external concentration, and the affected barrier. Neither efflux nor hyperporination contributed to ciprofloxacin tolerance of *E. coli* and *K. pneumoniae*. In the case of tobramycin, hyperporination of *E. coli* cells led to faster cell killing, whereas both efflux and the OM barrier contributed to *K. pneumoniae* persistence.

## DISCUSSION

*E. coli* and *K. pneumoniae* are closely related *Enterobacteriaceae* species that often occupy the same niche in human bodies and cause a similar range of infections (51). In this study, we found that the permeability barriers of the two species differ in their contributions to antibiotic susceptibility and persistence. The major attributes of their cell envelopes are very similar. Both species produce general porins located in the OM that facilitate non-specific diffusion of nutrients and some antibiotics (16). The most abundant porins (OmpF and OmpC) in *E. coli* and their respective OmpK35 and OmpK36 homologs in *K. pneumoniae* (Table S1) have very similar structures and conductivities, with OmpF/OmpK35 creating larger pores than OmpC/OmpK36 (17, 52). As might be expected from the size of the pores, molecules including hydrophilic antibiotics have higher permeability through the OmpF/OmpK35 porins than through OmpC/OmpK36. Indeed, except for macrolides and some sulfonamides, susceptibilities of tested antibiotics were only weakly affected by the OM barriers in either of the species, suggesting that most of the antibiotics use general porins to get access into cells (Fig. 3C). However, large antibiotics such as macrolides that are excluded by porins vary in the extent to which the OM barrier contributes to their antibacterial activities. We found that for some macrolides, the *K. pneumoniae* OM presented a more formidable permeation barrier than *E. coli*, pointing to porin-independent differences in properties of these barriers. Unlike *E. coli* BW25113, which is a derivative of K-12 and cannot produce O-polysaccharide chains of LPS due to a mutation (53), *K. pneumoniae* ATCC 43816 produces an intact LPS and a capsule. Extracellular polysaccharides forming a water-saturated mesh could potentially slow down diffusion of small molecules (54) but are not expected to limit intracellular penetration. However, differences in uptake pathways, if such are present, could contribute to species-specific differences in OM permeability barriers.

Both species produce highly homologous AcrAB-TolC efflux pumps that are constitutively expressed but are also regulated by local and global transcriptional regulators in response to antibiotics and various stresses (55). In this study, we compared the Δ*tolC* mutants of *E. coli* and *K. pneumoniae*, lacking the OM channel TolC, which is required for the activities of AcrAB-TolC and other RND efflux pumps in these species (Table S2). The exception is *K. pneumoniae* EefABC, which contains its own OM channel EefC and is homologous to a cryptic efflux pump in *Enterobacter aerogenes* (30). Interestingly, for most of the tested antibiotics, the effect of efflux on their antibacterial activities was strong but also species specific. Macrolides and tetracyclines were more strongly affected by Δ*tolC* mutation in *K. pneumoniae* than in *E. coli* (Fig. 3D). Since these differences were specific only to some of the representatives of these antibiotic classes, this result points to possible differences in substrate specificities of TolC-dependent efflux pumps or different levels of their expression. Possible contributions of species-specific efflux pumps acting across the inner membrane cannot also be excluded.

The dramatic effect of the growth media on efflux and OM contributions in antibacterial activities of antibiotics could possibly suggest some mechanisms underlying these species-specific differences (Fig. 4). For *E. coli*, the contribution of efflux (P/PE) in antibacterial activities was notably stronger in the rich LB broth than in the minimal M9 medium and affected most tested antibiotics (Fig. 4). This result suggests that efflux pumps are either downregulated or less effective in the minimal medium. Indeed, in one of the most systematic transcriptomics studies (45), the abundances of *E. coli acrA* and *acrB* decreased under nutrient downshift conditions, whereas the expression of the largest OmpF porin was found to be dramatically elevated. Perhaps a combination of both the lower efflux activity and the increased permeability of OM could be responsible for the decreased efflux effect on the antibiotic susceptibility of *E. coli* grown in M9. In addition, the growth rate of *E. coli* in M9 is two to three times slower than that in LB broth, which could also contribute to apparent differences in efflux contribution between these two media (56). The changes in antibiotic susceptibilities of *K. pneumoniae* were more complicated, with some antibiotics such as TET and FQ being more efficient in the rich LB medium and others not affected by differences in nutrients (Fig. 4). The transcripts of *K. pneumoniae acrAB* were also repressed during the nutritional downshift, but unlike in *E. coli*, no changes in the expression of OmpK35/K36 porins were found (45). The lack of correlation between the total barrier ratios of *E. coli* and *K. pneumoniae* in different media further shows that changes in the growth media do not affect the permeation of all antibiotics in the same way, and the mechanism of action of antibiotics and specific physicochemical properties of each compound play a major role.

The species-specific differences in the permeability barriers also contribute to the differences in antibiotic persistence. Although diverse mechanisms are proposed to contribute to the appearance of persisters, the most common outcome of their actions is the decrease in energy-generating components, leading to low proton-motive force and ATP, metabolism, and target activities (47, 57). Assuming that persistence is driven by a combination of stochastic and responsive mechanisms, Δ*tolC* mutations and hyperporination of *K. pneumoniae* and *E. coli* cells could lead to different numbers of the pre-existing persisters and those induced by the antibiotic stress. Our results show that in the case of ciprofloxacin, which induces DNA damage stress, the permeability barriers of *K. pneumoniae* and *E. coli* do not contribute to persistence when strains are exposed to the same external drug concentration of this antibiotic (Fig. 5). However, tobramycin, which induces the cell envelope stress, was more effective in killing cells with compromised permeability barriers. Surprisingly, the external concentration of ciprofloxacin was important for the *E. coli* tolerance, but the rate of cell killing did not correlate with the strain-specific MICs of ciprofloxacin. It seems likely that Δ*tolC* mutations and hyperporination do not affect the pre-existing population of persisters but are important for persistence induced by specific stresses possibly related to the mechanism of action of the antibiotic. Lastly, the constructed double-compromised *K. pneumoniae* strain, which is hypersusceptible to various antibiotics and the four-strain set with variable permeability barriers, can be used during drug discovery and optimization campaigns targeting carbapenem- and multidrug-resistant *Enterobacteriaceae.*

## MATERIALS AND METHODS

### Strains and growth conditions

LB broth (10 g of Bacto tryptone, 5 g of yeast extract, and 5 g of NaCl per liter, pH 7.0) or LB agar (LB broth with 15 g of agar per liter) were used for bacterial growth. M9-MOPS minimal medium supplemented with 0.2% xylose and 0.5% sodium citrate was used in antibiotic susceptibility experiments (12). When indicated, cultures were induced with 0.5% and 0.1% L-arabinose (ARA) for *K. pneumoniae* and *E. coli* strains, respectively, to induce the expression of the pore. *K. pneumoniae* ATCC 43816 metabolizes ARA; hence, the higher concentration of this inducer was needed to produce and maintain the levels of hyperporination. Immunoblotting (see below) showed that at 0.5% ARA, the expression levels of the pore remained similar during 24 h of growth (Fig. S4). For strain selection, gentamicin (15 or 30 µg/mL), kanamycin (25 µg/mL), and chloramphenicol (25 or 10 µg/mL) were used.

### MIC determination

The MIC of selected antibiotics against *K. pneumoniae* strains (Table 2) was determined as described previously (31). The strains were induced with 0.5% ARA.

Susceptibility to different classes of antibiotics: 10 SULFAs, 8 FQs, 11 MACs, 7 TETs, 4 PHEs, 7 CEFs and a single representative of oxazolidinone, linezolid, mupirocin (pseudomonic acid), and vancomycin were determined by a twofold broth dilution method, as described in reference 12, with a few modifications. Briefly, exponentially growing cells were inoculated at a density of 10^5^ cells/mL into wells containing LB or M9-MOPS medium with appropriate inducer in the presence of twofold increasing concentrations of drugs under investigation. Thermo Scientific Multidrop Combi was used for medium and cell distribution into 96-well plates. Serial dilution of antibiotics in the medium was carried out by Tecan FREEDOM EVO Liquid Handler. Cell growth was determined visually, and the optical density (OD) was measured by Tecan Spark 10M Plate Reader after incubation of the microtiter plates at 37°C for 20 h. For the determination of IC_50_ values, the growth curves were fitted to a Hill equation (58). All experiments were done with at least two biological replicates and two technical repeats. IC_50_ values were averaged, and standard deviation was calculated.

### Construction of plasmids and strains

Bacterial strains and plasmids used in this study are listed in Table 1.

To delete *tolC* from the ATCC 43816 strain, a modified α-Red recombination protocol (36) was used. Since the strain is resistant to β-lactams, we replaced the *bla* gene in pKD46 with the kanamycin resistance gene (Km) from pGK-R6K-mini-Tn*7*T-*araC*-P_BAD_-MCS by using Gibson assembly cloning (Invitrogen, Thermo Fisher Scientific). The pIL155 (pKD46-Km) plasmid was electroporated to *K. pneumoniae* ATCC 43816 (Kp) and selected on kanamycin (25 µg/mL). Kp(pIL155) was subcultured (1:20) from the overnight culture, induced and grown at 30°C until the OD_600_ reached 0.6. Electrocompetent cells were prepared by washing three times in ice-cold water. PCR product (0.5 µg) was added to 10× concentrated electrocompetent cells. A PCR product (∆*tolC*::Gm) was amplified from the constructed pIL154 plasmid. To create the plasmid, 1 kb DNA fragments located upstream and downstream of the *tolC* gene were amplified from the genomic DNA of ATCC 43816 and incorporated into the plasmid by Gibson assembly. Positive colonies were selected on LB agar plates containing gentamicin (15 µg/mL). The remaining steps were according to the published one-step inactivation protocol in *E. coli* (36). After removing the gentamicin resistance cassette, the gene deletion was confirmed by PCR.

### Insertion of *FhuA ∆C/∆4L* into *K. pneumoniae* chromosome

A mini-Tn*7*T-based protocol with some modification was used to insert *fhuA*Δ*C/*Δ*4L* into the Kp chromosome (34). Helper plasmid pTNS3 and suicide vectors pGK-R6K-mini-Tn*7*T-*araC*-P_BAD_-MCS (Km^r^) and pGK-R6K-mini-Tn*7*T-*araC*-P_BAD_-*fhuA*Δ*C/*Δ*4L* (Km^r^) were transformed into RHO3 (SM10 dap^−^ λpir^+^) *E. coli* cells. Mini Tn*7* cassettes were integrated onto the chromosome of Kp and Kp ∆TolC by triparental mating. We patched together the *E. coli* donor strain (RHO3-pGK-R6K-mini-Tn*7*T-*araC*-P_BAD_-MCS or RHO3-pGK-R6K-mini-Tn*7*T-*araC*-P_BAD_-*fhuA*Δ*C/*Δ*4L*), the *E. coli* helper strain (RHO3-pTNS3), and the *K. pneumoniae* recipient strain (Kp or IL205) on an LB plate supplemented with 0.3 mM 2,6-diaminopimelic acid and grew them for 20 h at 37°C. Selection was carried out by growing strains on LB agar containing kanamycin (25 µg/mL) and incubating them for 16 h at 37°C. The insertions were confirmed by PCR.

### Vancomycin spot assay

To determine the susceptibility to vancomycin, 100 µL of overnight grown cells was added to 4 mL of soft LB agar (55°C) containing 0.5% ARA. The suspension was poured onto LB agar plates containing the same concentration of the inducer, and the spot assay was carried out as described before (31). The plates were then incubated in an upright position at 37°C for 20 h and scanned, and zones of inhibition were measured.

Vancomycin-resistant colonies found in this experiment were isolated and screened for susceptibility to rifampicin, carbenicillin, and vancomycin. The presence of the *araC*-P_BAD_-*fhuA*Δ*C/*Δ*4L* (Km^r^) insertion in the Tn7 sites was confirmed by PCR, and the entire insertion fragments from five Kp-P and four Kp-PE colonies were amplified from chromosomes and sequenced by Plasmidsaurus Inc.

### Pore expression and analysis

To confirm the expression of the pore, the overnight cultures of IL203, IL204, IL206, and IL207 cells were subcultured 1:50 into a 100 mL fresh LB broth, induced with 0.5% arabinose ARA, and grown at 37°C with aeration for 6 h. Cells were harvested, lysed, and membrane fractions were solubilized and enriched as described before (38). Eluted fractions were boiled for 5 min in the reducing SDS-sample buffer and analyzed by SDS-PAGE followed by immunoblotting with primary monoclonal anti-6xHis antibodies (Sigma) followed by a secondary alkaline phosphatase-conjugated anti-mouse immunoglobulin antibody (Sigma). 5-Bromo-4-chloro-3-indolylphosphate and nitroblue tetrazolium were used to visualize the bands.

### Hoechst 33342, ethidium, and NPN intracellular uptake assays

Overnight cultures were diluted 1:100 into fresh LB medium and grown at 37°C up to OD_600 =_ 0.3. The cells were then induced with 0.5% or 0.1% ARA for *K. pneumoniae* and *E. coli*, respectively, and incubated for 3 h. Cells were collected by centrifugation at 4,000 rpm for 20 min at room temperature, and the pellet was resuspended in 50 mM HEPES-KOH buffer (pH 7.0) containing 1 mM magnesium sulfate and 0.4 mM glucose (HMG buffer) and pelleted at 4,000 rpm at room temperature. The pellet was resuspended in HMG buffer and adjusted to OD ~1.0 and kept at room temperature during the experiment.

The uptake assay was performed as described before (38, 40) in a temperature-controlled microplate reader (TECAN SPARK 10M Microplate Reader) equipped with a sample injector, in a fluorescence mode. Fluorescence of Hoechst was followed at *λ*_ex_ = 355 nm and *λ*_em_= 450 nm at a gain of 75, EtBr – at *λ*_ex_ = 480 nm and *λ*_em_= 610 nm at a gain of 65, and NPN – at *λ*_ex_ = 350 nm and *λ*_em_= 405 nm at a gain of 65. All measurements were done two or three times in duplicate. This data were fitted in MATLAB (MathWorks) to a simple exponential equation in the form of *F* = *A*1 + *A*2[1 − exp(−kt)] (40).

### Ciprofloxacin and tobramycin persistence experiments

Bacteria were inoculated at 1:100 into LB medium from an overnight culture. Cell cultures were incubated for 1 h, then 0.5% (Kp) or 0.1% ARA (Ec) was added to induce the expression of pore. After overnight incubation, cells were washed in fresh LB medium and diluted to OD_600_ ~1.0 and incubated at 37°C with aeration with tobramycin or ciprofloxacin at desired concentrations. To quantify persisters, 1 mL of cells was removed and pelleted by centrifugation in 0, 1, 3, 6, 8, and 24 h of incubation with the drug. The pellets were washed twice with 1 mL of sterile 1% NaCl and resuspended with 1 mL of sterile 1% NaCl. Cells were serially diluted in 1% NaCl and spotted onto LB agar. The plates were incubated at 37°C for 16 h. Dilutions that enabled 5–30 colonies to be counted were used for CFU enumeration.

## SUPPLEMENTAL MATERIAL

Supplemental material aac.00085-25-s0001.docx Tables S1 and S2; Fig. S1 to S4.

Table S3 aac.00085-25-s0002.xlsx MIC and IC50 values of antibiotics in *K. pneumoniae* and *E. coli* strains.

ASM does not own the copyrights to Supplemental Material that may be linked to, or accessed through, an article. The authors have granted ASM a non-exclusive, world-wide license to publish the Supplemental Material files. Please contact the corresponding author directly for reuse.
