# Supplementary material for: No two are alike: on the role of Klebsiella pneumoniae permeability barriers in antibiotic susceptibility and persistence
Source: Antimicrob Agents Chemother. 2025 Jun 17;69(8):e00085-25. doi: 10.1128/aac.00085-25 (PMC12327008; doi:10.1128/aac.00085-25)
Supplement: Supplemental material — Tables S1 and S2; Fig. S1 to S4. [file aac.00085-25-s0001.docx]

**Supplemental materials for:**

**No two are alike: the role of *Klebsiella pneumoniae* permeability barriers in antibiotic susceptibility and persistence**

Inga V. Leus and Helen I. Zgurskaya*

University of Oklahoma, Department of Chemistry and Biochemistry, Norman, OK, USA

* Corresponding author: [elenaz@ou.edu](mailto:elenaz@ou.edu)

**Table S1. Porins of *K. pneumoniae* ATCC 43816 implicated in antibiotic permeation and their homologs in *E. coli.***

| Porin in *K. pneumoniae* | Gene  (ATCC 43816) | *E. coli* homologs | Reference |
| --- | --- | --- | --- |
| OmpK35 | IT767_10690 | OmpF | (1-3) |
| OmpK36 | IT767_01530 | OmpC | (1-3) |
| OmpK37 | IT767_08355 | OmpN | (3, 4) |
| OmpK38(OmpC^a^) | IT767_03145 | - | (3) |
| PhoE | IT767_14360 | OmpE | (3) |
| OmpK26 | IT767_24180 | - | (3, 5) |

^a^, annotated as OmpC in NCBI.

**Table S2. Three-component RND efflux pumps of *K. pneumoniae* ATCC 43816 and their substrate specificities.**

| Name | MFP | RND | OM channel | Substrates | Reference |
| --- | --- | --- | --- | --- | --- |
| AcrAB | IT767_13515 | IT767_13520 | TolC-IT767_23030 | β-lactams, Macrolides, Fluoroquinolones, Novobiocin, Tetracycline, EtBr, Acriflavine, SDS, HT, Rhodamine | (6) |
| AcrD (KexC) | - | IT767_00640 | TolC | β-lactams, Novobiocin, SDS | (7) |
| OqxAB | IT767_25625 | IT767_25620 | TolC | Quinolone,  β-lactams,  Novobiocin, EtBr,  Acriflavine, SDS, HT, Rhodamine | (8, 9) |
| EefABC | IT767_20060 | IT767_20055 | IT767_20050 | β-lactams, Macrolides, Novobiocin, Tetracycline, EtBr, Acriflavine, SDS, HT, Rhodamine | (10) |
| MdtABC (KexVWX) | IT767_02090 | IT767_02085  IT767_02080 | TolC | β-lactams, Novobiocin | (7) |
| KexEF | IT767_21930 | IT767_21925 | TolC | β-lactams, Macrolides, Novobiocin, SDS, Tetracycline, EtBr, HT, Rhodamine | (7) |
| KexTU | IT767_12910 | IT767_12905 | TolC | unknown | (7) |
| KexJK | IT767_04640 | IT767_04645 | TolC | SDS | (7) |
| KexSR | IT767_11745 | IT767_11750 | TolC | unknown | (7) |

Figure S1. Kinetics of Ethidium Bromide (EtBr) uptake into Kp (A-D) and Ec (E-H) strains with different efflux capacities and OM permeabilities. Exponentially growing cells were incubated with strain specific concentration of arabinose for 3 hours and added to buffered glucose solutions containing increasing from 1 µg/ml to 16 µg/ml concentrations of EtBr. Each time point represents the average of at least two biological replicates with two technical repeats ± standard deviation (SD).


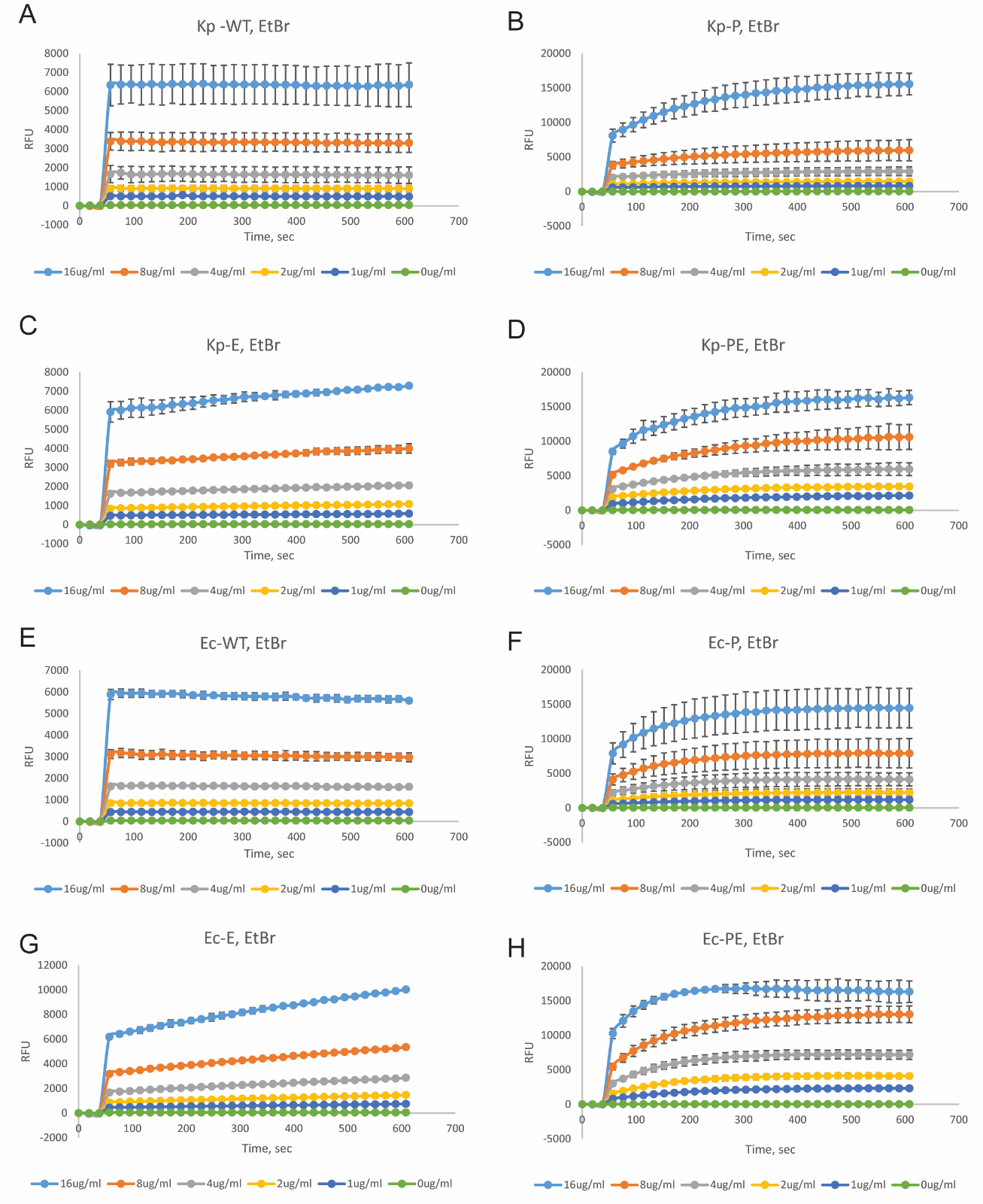


Figure S2. Kinetics of HT uptake into Kp (A-D) and Ec (E-H) strains with different efflux capacities and OM permeabilities. Exponentially growing cells were incubated with 0.5% or 0.1% ARA for 3 hours and added to buffered glucose solutions containing increasing from 1 µM to 16 µM concentrations of HT. Each time point represents the average of at least two biological replicates with two technical repeats ± standard deviation (SD).


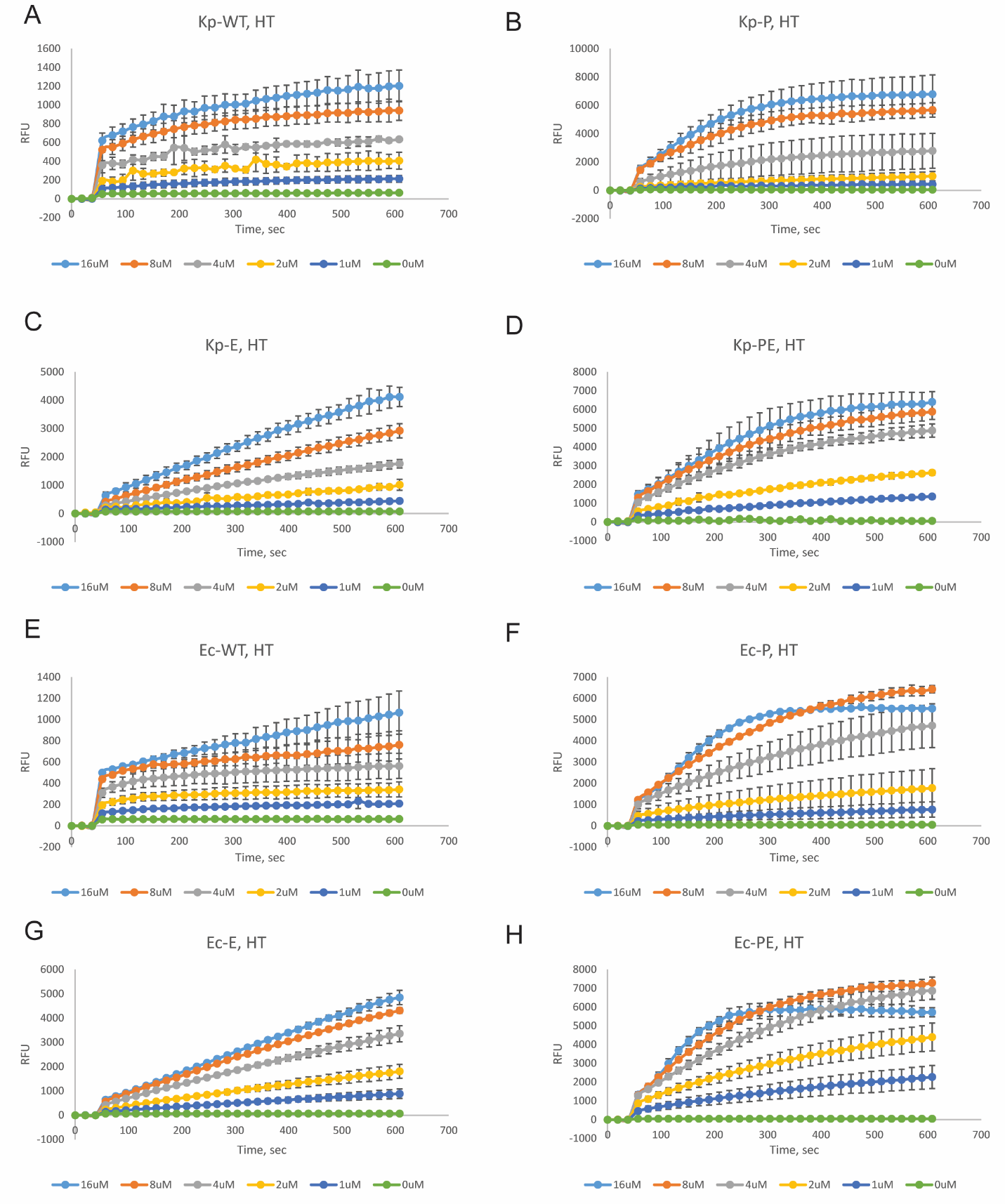


Figure S3. Kinetics of NPN uptake into Kp (A-D) and Ec (E-H) strains with different efflux capacities and OM permeabilities. Data represents real-time kinetics of changes in NPN fluorescence (1 µM-16 µM final external concentration). Each time point is the average of at least two biological replicates with two technical repeats ± standard deviation (SD).


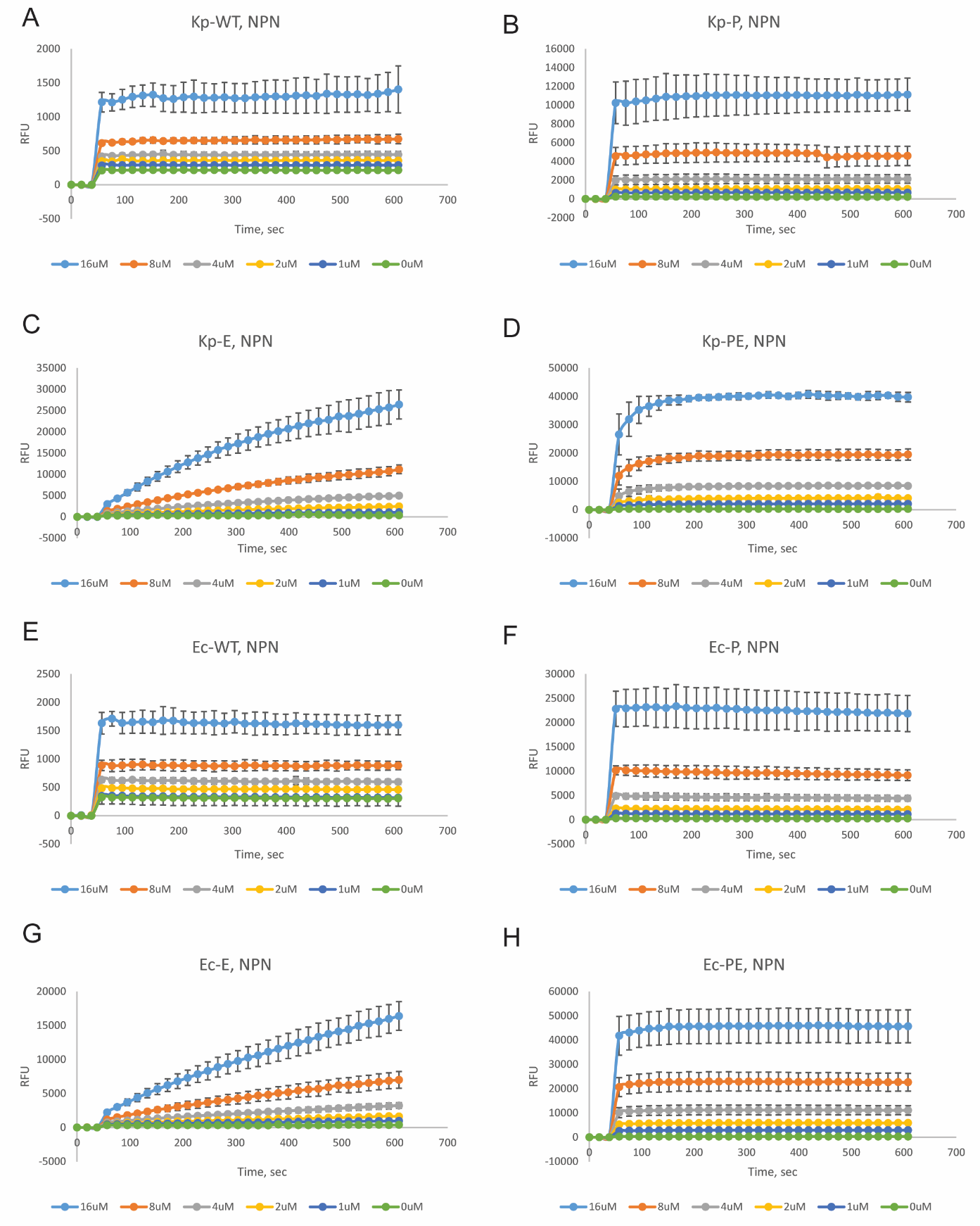


Figure S4. Expression of the Pore during bacterial growth. Kp-P cells were grown as described in Methods, cell aliquots were collected in 3, 6 and 24 h after the induction with 0.5% ARA. The Pore protein was visualized by immunoblotting of solubilized and partially purified OM proteins with anti-His antibody (Sigma).


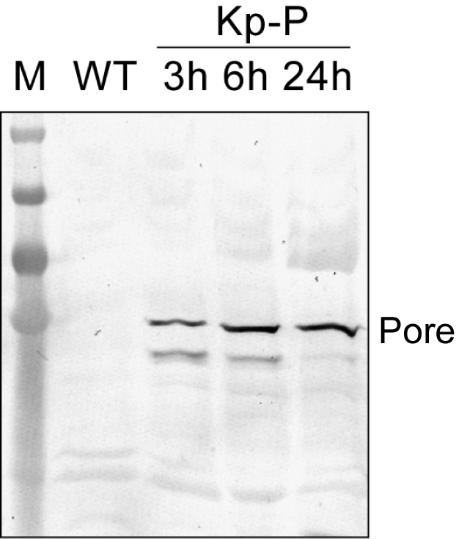


1. Tsai YK, Fung CP, Lin JC, Chen JH, Chang FY, Chen TL, Siu LK. 2011. Klebsiella pneumoniae outer membrane porins OmpK35 and OmpK36 play roles in both antimicrobial resistance and virulence. Antimicrob Agents Chemother 55:1485-93.

2. Sugawara E, Kojima S, Nikaido H. 2016. Klebsiella pneumoniae Major Porins OmpK35 and OmpK36 Allow More Efficient Diffusion of β-Lactams than Their Escherichia coli Homologs OmpF and OmpC. J Bacteriol 198:3200-3208.

3. Rocker A, Lacey JA, Belousoff MJ, Wilksch JJ, Strugnell RA, Davies MR, Lithgow T. 2020. Global Trends in Proteome Remodeling of the Outer Membrane Modulate Antimicrobial Permeability in Klebsiella pneumoniae. mBio 11.

4. Doménech-Sánchez A, Hernández-Allés S, Martínez-Martínez L, Benedí Vicente J, Albertí S. 1999. Identification and Characterization of a New Porin Gene of Klebsiella pneumoniae: Its Role in β-Lactam Antibiotic Resistance. Journal of Bacteriology 181:2726-2732.

5. García-Sureda L, Doménech-Sánchez A, Barbier M, Juan C, Gascó J, Albertí S. 2011. OmpK26, a Novel Porin Associated with Carbapenem Resistance in Klebsiella pneumoniae. Antimicrobial Agents and Chemotherapy 55:4742-4747.

6. Li DW, Onishi M, Kishino T, Matsuo T, Ogawa W, Kuroda T, Tsuchiya T. 2008. Properties and expression of a multidrug efflux pump AcrAB-KocC from Klebsiella pneumoniae. Biol Pharm Bull 31:577-82.

7. Ni RT, Onishi M, Mizusawa M, Kitagawa R, Kishino T, Matsubara F, Tsuchiya T, Kuroda T, Ogawa W. 2020. The role of RND-type efflux pumps in multidrug-resistant mutants of Klebsiella pneumoniae. Scientific Reports 10:10876.

8. Rodríguez-Martínez JM, Díaz de Alba P, Briales A, Machuca J, Lossa M, Fernández-Cuenca F, Rodríguez Baño J, Martínez-Martínez L, Pascual Á. 2013. Contribution of OqxAB efflux pumps to quinolone resistance in extended-spectrum-β-lactamase-producing Klebsiella pneumoniae. J Antimicrob Chemother 68:68-73.

9. Kim HB, Wang M, Park CH, Kim EC, Jacoby GA, Hooper DC. 2009. oqxAB encoding a multidrug efflux pump in human clinical isolates of Enterobacteriaceae. Antimicrob Agents Chemother 53:3582-4.

10. Coudeyras S, Nakusi L, Charbonnel N, Forestier C. 2008. A tripartite efflux pump involved in gastrointestinal colonization by Klebsiella pneumoniae confers a tolerance response to inorganic acid. Infection and immunity 76:4633-4641.
